# Investigation of Trypanothione Reductase as a Drug Target in *Trypanosoma brucei*

**DOI:** 10.1002/cmdc.200900262

**Published:** 2009-11-18

**Authors:** Daniel Spinks, Emma J Shanks, Laura A T Cleghorn, Stuart McElroy, Deuan Jones, Daniel James, Alan H Fairlamb, Julie A Frearson, Paul G Wyatt, Ian H Gilbert

**Affiliations:** Drug Discovery Unit, College of Life Sciences, University of Dundee, Sir James Black CentreDundee, DD1 5EH (UK), Fax: (+44)1382-386-373E-mail: i.h.gilbert@dundee.ac.uk

**Keywords:** human African trypanosomiasis, pyrimidopyridazines, quinolines, *trypanosoma brucei*, trypanothione reductase

## Abstract

There is an urgent need for new drugs for the treatment of tropical parasitic diseases such as human African trypanosomiasis, which is caused by *Trypanosoma brucei*. The enzyme trypanothione reductase (TryR) is a potential drug target within these organisms. Herein we report the screening of a 62000 compound library against *T. brucei* TryR. Further work was undertaken to optimise potency and selectivity of two novel-compound series arising from the enzymatic and whole parasite screens and mammalian cell counterscreens. Both of these series, containing either a quinoline or pyrimidinopyrazine scaffold, yielded low micromolar inhibitors of the enzyme and growth of the parasite. The challenges of inhibiting TryR with druglike molecules is discussed.

## Introduction

Human African trypanosomiasis (HAT) is a serious health problem in sub-Saharan Africa, with an estimated 50000 new cases each year. HAT, a fatal disease unless treated, is caused by the protozoan parasites *Trypanosoma brucei gambiense* and *T. b. rhodesiense*, which initially reside within the blood stream and then subsequently penetrate into the central nervous system giving rise to the classical symptoms of HAT. The current drugs to treat HAT are inadequate due to poor efficacy, side effects and the requirement for parenteral administration, which is not appropriate for a rural African setting.^1^,^2^

**Figure 1:**
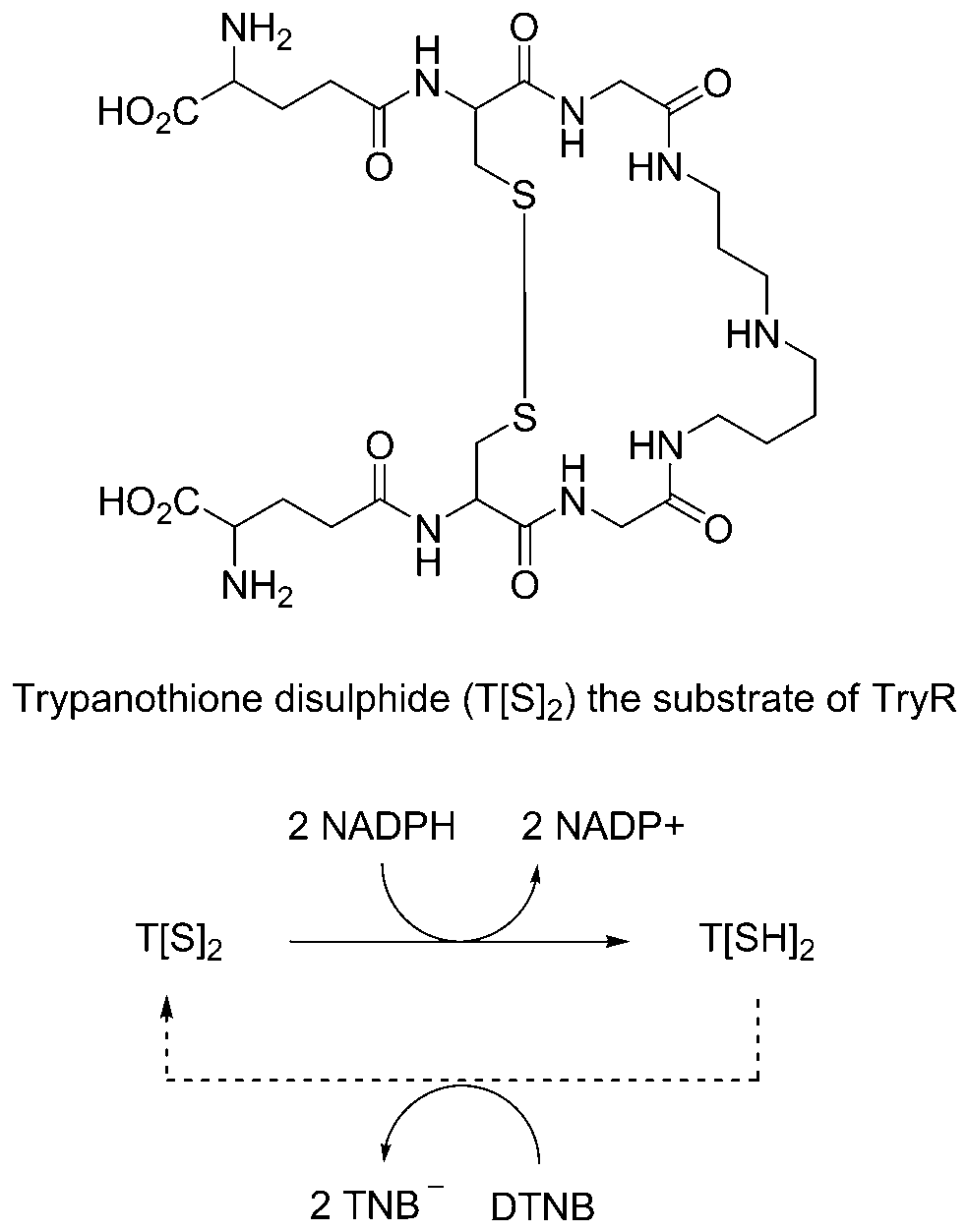
The structure of trypanothione and the principle of the DTNB-coupled assay for TryR.

Herein we report a target-based approach to the discovery of novel inhibitors of trypanothione reductase (TryR) as a potential therapy for HAT. TryR is an NADPH-dependent flavoprotein disulphide oxidoreductase unique to, and essential for growth of, trypanosomatid parasites, whose function is to convert trypanothione disulphide (*N*^1^,*N*^8^-*bis*(glutathionyl)spermidine disulphide, T[S]_2_; Figure 1) into the physiologically relevant reduced dithiol (T[SH]_2_).^3^ In these parasites, T[SH]_2_ serves as a substitute for many of the metabolic and antioxidant functions ascribed to glutathione (GSH) in mammalian cells.^4^ Mammalian glutathione reductase (GR) is the nearest homologue to TryR. However, the host and parasite enzymes have significant differences in their active site architecture resulting in a pronounced ability to discriminate between their respective disulphide substrates. These features make TryR an attractive target for selective drug design.^5^

Before carrying out a drug discovery programme TryR was assessed for appropriateness using the traffic light scoring system that we recently described.^6^ The process assesses a target in terms of: level of validation (genetic and chemical), assay feasibility, potential for toxicity and resistance, druggability, and level of structural information available allowing cross target comparisons and prioritisation for entry into hit discovery programmes. The results of this exercise are illustrated in Table 1. In brief, there was sufficient supporting information in the areas of genetic and chemical validation, assay feasibility and protein structure to progress the target into hit discovery. The main area for caution for this target was druggability since the active site is rather large and featureless.

**Table 1:** Antiparasitic drug target evaluation for trypanothione reductase.

Criteria	Status	Comments
Assay feasibility	green	Recombinant enzyme available in large quantities. 96 well assay in place
Genetic/chemical validation	green	Target essential in trypanosomes as evidenced by conditional knock-out.^3^ Some chemical validation from inhibitors such as trivalent antimonials and melaminophenyl arsenicals.
Druggability	amber	Several published low micromolar low molecular weight inhibitors. The active site of TryR is large and therefore the identification of potent low molecular weight inhibitors could be an issue.
Potential for resistance	amber	TryR is a single copy gene in *T. brucei* and no reported gene amplification in response to inhibitors has been reported. Reversal of competitive inhibitors by accumulation of T[S]_2_ is a potential liability
Potential for toxicity	green	Significant differences in substrate specificity and structure of TryR to corresponding human homologue, glutathione reductase.
Protein structural information	green	Crystal structures of recombinant *T.cruzi* TryR, alone and in combination with substrates and a covalently linked inhibitor

A number of different TryR inhibitors have been reported in the literature. These can be broadly classified as tricyclics,^7^ polyamine analogues,5f,h,^8^ redox inhibitors,^9^ substrate analogues,^10^ and compounds identified through screening of a library of druglike compounds.^11^ To our knowledge, none of these series have progressed beyond the early discovery phase.

## Results and Discussion

### Hit discovery

In order to discover novel lead compounds against TryR, a high-throughput screen of an in-house designed diverse compound library^12^ (62000 compounds) was performed.

A nonenzymatically coupled colourimetric method for detecting TryR activity as developed by Fairlamb et al.^13^ was employed. In this assay, the activity of TryR is coupled to the reduction of DTNB (5,5′-dithiobis[2-nitrobenzoic acid]) by T(SH)_2_ to form the yellow thionitrobenzoate anion (TNB^−^) (see Figure 1). Unlike the direct assay, this method increases assay sensitivity, and allows the assay to proceed in a linear fashion for extended time periods with T[S]_2_ concentrations at or below *K*_m_.

The TryR screening assay was miniaturised to a 384-well plate format and optimised to the standard required to support a large-scale screening campaign. The assay was assessed for robustness in an automated environment yielding the following typical performance statistics: Z′=0.84±0.001; %CV (plate)=3.65±0.4; S:B=10±0.25; IC_50_ (clomipramine)=12.4±0.14 μm. Clomipramine was used as a standard throughout the screening process.

**Figure 2:**
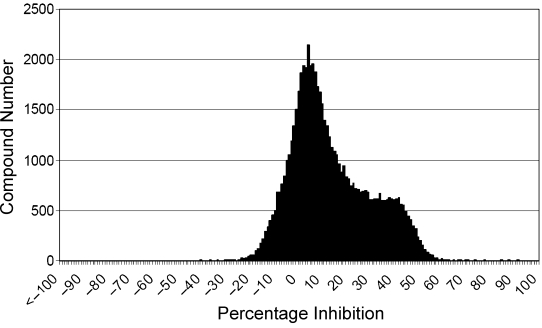
Single-point screen percentage inhibition (PI) distribution against TryR for all compounds returning PI values ≥50 (*n*=901).

TryR was initially screened in single-point mode at 30 μm and the percentage inhibition (PI) value calculated for each compound (Figure 2). The distribution of hits deviated from normality due to a shoulder of activity between 20 and 50 PI, indicating a capacity of TryR to bind with a low affinity to a range of diverse structures. A statistical assessment of the screen relative to the error around the full signal controls (5× standard deviations) indicated that PI values ≥42% should be considered as highly significant. All compounds yielding PI ≥50% were therefore selected for re-testing in duplicate (30 μm). A total of 767 compounds were confirmed as hits giving a confirmation rate (primary to re-test screen) of 84% and an overall confirmed hit rate of 1.24%.

**Figure 3:**
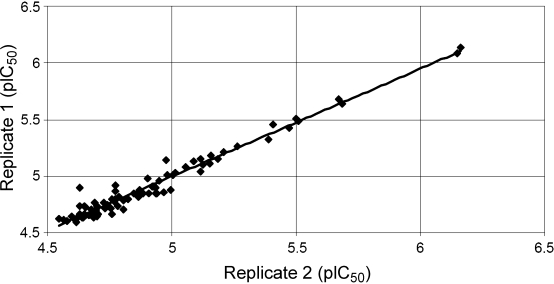
pIC_50_ correlation between two replicate assays.

A preliminary chemistry assessment of the confirmed hits identified a selection of 120 compounds for potency determinations. Each compound was subjected to a ten-point half-log titration in duplicate ranging from 30 μm to 1 nm. In all cases, residual material from the potency compound plates was subjected to LCMS analysis for structure and purity confirmation. Figure 3 illustrates the ability of this assay to report reliable potency values for test compounds, yielding a correlation coefficient (*R*^2^) value of 0.967 between replicate pIC_50_ determinations.

Analysis of the potency data for the best hits revealed multiple putative hit series covering a range potencies from 0.7 to 30 μm. Key compounds were resynthesised or resupplied to further confirm their activity and identity. From the five series initially identified, two were deemed of sufficient interest to progress into formal hit validation. The aim of this phase was to rapidly assess the existence of defined structure–activity relationships (SAR), whilst securing a significant increase (>10-fold) in activity over the initial hit compound.

### Hit series 1

Series 1 was based on a quinoline scaffold, which afforded a number of possible points of variation: the 5-methylfuran in the 2-position, the amide in the 4-position, and the 6-bromo substituent. To expand the SAR, we prepared or purchased 84 analogues. Table 2 provides data on the most potent and significant inhibitors.

**Table 2:** Activity of series 1 (compounds **1**–**26**) against TryR.

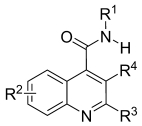					
Compd	R^1^	R^2^	R^3^	R^4^	TryR IC_50_ [μm]
1	CH_2_CH_2_NMe_2_	6-Br	5′-Methyl-furan-2-yl	H	1.7
2	CH_2_CH_2_NHMe	6-Br	5′-Methyl-furan-2-yl	H	1.1
3	CH_2_CH_2_NMe_3_+	6-Br	5′-Methyl-furan-2-yl	H	1.2
4	CH_2_CH_2_-*N*-morpholine	6-Br	5′-Methyl-furan-2-yl	H	4.2
5	2-pyridyl methyl	6-Br	5′-Methyl-furan-2-yl	H	4.7
6	3-pyridyl	6-Br	5′-Methyl-furan-2-yl	H	7.2
7	CH_2_CH_2_CH_2_-*N*-imidazole	6-Br	5′-Methyl-furan-2-yl	H	5.1
8	CH_2_CH_2_NMe_2_	6-Cl	5′-Methyl-furan-2-yl	H	3.1
9	CH_2_CH_2_NMe_2_	7-Br	5′-Methyl-furan-2-yl	H	5.7
10	CH_2_CH_2_NMe_2_	8-Br	5′-Methyl-furan-2-yl	H	5.8
11	2-pyridyl methyl	H	5′-Methyl-furan-2-yl	H	12.1
12	CH_2_CH_2_NMe_2_	H	5′-Methyl-furan-2-yl	H	14.4
13	CH_2_CH_2_NMe_2_	6-Cl	4′-Methylphenyl	CH_3_	26.5
14	CH_2_CH_2_-*N*-morpholine	H	5′-Methyl-furan-2-yl	H	100
15	CH_2_Ph	6-Br	5′-Methyl-furan-2-yl	H	>100
16	CH_2_CH_2_ CH_2_-*N*-morpholine	6-Cl	5′-Methyl-furan-2-yl	H	11.6
17	CH_2_CH_2_NMe_2_	6-Br	3′-Methoxyphenyl	H	>100
18	CH_2_CH_2_NMe_2_	6-Br	Phenyl	H	>100
19	CH_2_C_6_H_4_-p-SO_2_NH_2_	6-Br	5′-Methyl-furan-2-yl	H	8.4
20	CH_2_CH_2_NMe_2_	6-Br	Furan-2-yl	H	32.4
21	CH_2_CH_2_NMe_2_	6-Br	Pyrid-4-yl	H	96.3
22	CH_2_CH_2_NMe_2_	6-Br	5′-Methyl-thiophen-2-yl	H	>100
23	-*N*-morpholine	6-Br	5′-Methyl-furan-2-yl	H	>100
24	CH_2_-furan-2-yl	6-Br	5′-Methyl-furan-2-yl	H	>100
25	CH_2_CH_2_Ph	6-Br	5′-Methyl-furan-2-yl	H	>100
26	CH_2_CH_2_NMe_2_	6-F	5′-Methyl-furan-2-yl	H	>100

**Scheme 1:**
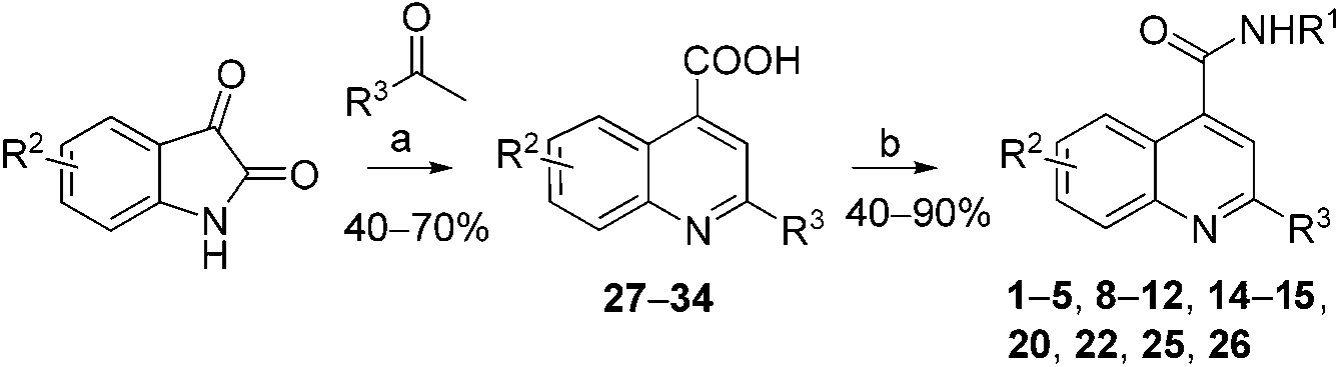
Synthetic route for hit series 1. *Reagents and conditions*: a) Ketone, KOH, EtOH/H_2_O, 110°C, 12 min, μW; b) R^1^NH_2_, CDI, CH_2_Cl_2_, RT, 18 h.

The general synthetic route is shown in Scheme 1. In brief, the indoline-2,3-dione was reacted with an appropriate ketone to give the quinoline. The carboxylic acid could then be condensed with appropriate amines to form the required amides.

**Figure 4:**
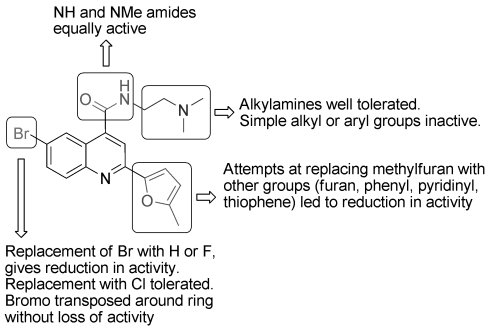
SAR for hit series 1.

A summary of the SAR is given in Figure 4. Essentially, any variation of the 5-methylfuran at the 2-position led to a decrease in activity (**1** and **17, 20** and **22**; **8** and **13**; **12** and **18**); in the 4-position, amides with a basic substituent, such as dimethylaminoethyl were more potent than neutral aryl or alkylaryl amides (**15, 23, 24, 25**); and the 6-bromo group could be moved around the ring without too much effect on the activity (**1**, **9**, **10**). Although replacing the 6-bromo substituent with a chloro atom led to a small effect on activity (**1** and **8**, **16**), replacement of the 6-bromo with hydrogen or fluorine led to loss in activity (**1** and **12**; **4** and **14**, **1** and **26**). Particularly with substituents on the 4-position, increases in MW and clog*P* led to small increases in potency, suggestive of a general surface contact between inhibitors and protein. There may be a similar effect with the 6-bromo group, as changes in location to the 7- or 8-position of the quinoline ring system, or even replacing it with a chlorine, led to only small effects on activity. It was difficult to deduce which changes, if any, would increase potency at these positions. It is possible that the 5-methylfuran at the 2-position is making a very specific hydrogen bonding and/or π-stacking interaction, which accounts for the requirement for this group at this position.

### Hit series 2

**Scheme 2:**
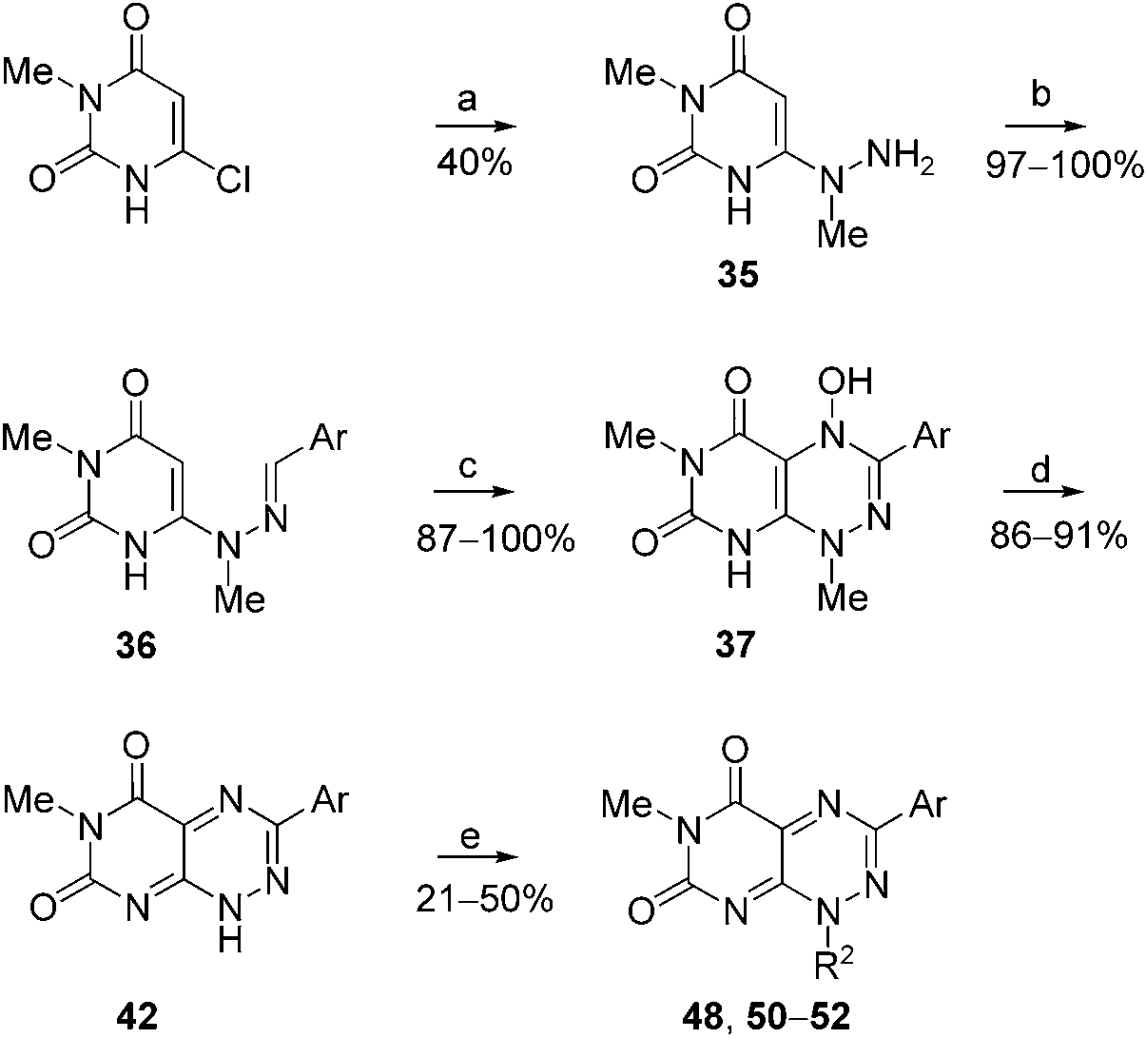
Synthetic Route for hit series 2. *Reagents and conditions*: a) H_2_NNHMe, EtOH, 80°C, 1 h; b) ArCOH, EtOH, 80°C, 2 h; c) NaNO_2_, AcOH, 0→20°C, 16 h; d) DMF, 90°C, 2 h; e) R^2^Br, Cs_2_CO_3_, 80°C, 3 h, DMF.

**Figure 5:**
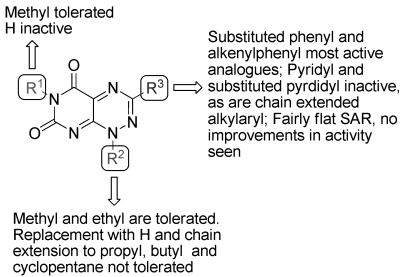
SAR for hit series 2.

Series 2, containing the pyrimidopyridazines scaffold, gave five compounds with an inhibition of >62% in the initial screen. The general synthetic route is outlined in Scheme 2. The substituted 6-chlorouracil starting material was made by condensation of the appropriately substituted urea with malonic acid, followed by chlorination. The chloride was displaced with an appropriate hydrazine. The hydrazine intermediate (**35**) was then condensed with aldehyde and cyclisation was achieved by treatment with sodium nitrite followed by dehydration through microwave heating in DMF with molecular sieves to give **42**.^14^ The free NH could be alkylated with various alkyl bromides to give the desired product. In total ∼30 compounds from this series were assayed. Table 3 provides data on the most potent and significant inhibitors. A summary of the SAR is given in Figure 5.

**Table 3:** Activity of series 2 (compounds **31**–**44**) against TryR.

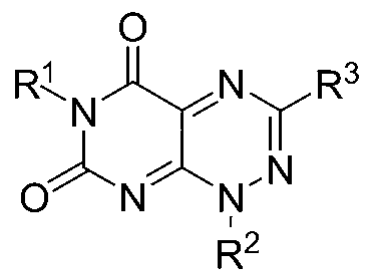				
Compd	R^1^	R^2^	R^3^	TryR IC_50_ [μm]
39	Me	Me	4′-Methylphenyl	8.6
40	Me	CH_2_CH_3_	Thiophen-2-yl	12.8
41	Me	CH_2_CH_3_	2′-Fluoro-5′-Bromophenyl	10.2
42	Me	H	2′-Fluoro-5′-Bromophenyl	>100
43	H	H	2′-Fluoro-5′-Bromophenyl	92
44	Me	CH_2_CH_3_	Pyridine-3-yl	>100
45	Me	CH_2_CH_3_	4′-Bromothiophen-2-yl	13.0
46	Me	CH_2_CH_3_	4′-Trifluoromethylphenyl	15.2
47	Me	CH_2_CH_3_	Phenyl	11.3
48	Me	CH_2_CH_2_NMe_2_	2′-Fluoro-5′-Bromophenyl	>100
49	Me	CH_2_CH_3_	CH=CHC_6_H_5_	2.6
50	Me	CH(CH_3_)_2_	2′-Fluoro-5′-Bromophenyl	>50
51	Me	(CH_2_)_3_CH_3_	2′-Fluoro-5′-Bromophenyl	>100
52	Me	cyclopentyl	2′-Fluoro-5′-Bromophenyl	>100

Small alkyl groups were required in positions R^1^ (methyl) and R^2^ (methyl and ethyl) as replacement of R^2^ or both R^1^ and R^2^ with hydrogen led to a loss in activity (**42** and **43**). The methyl at R^2^ could be replaced by a small alkyl group; however, replacement with larger alkyl groups led to a decrease in activity. A number of different aromatic substituents were tolerated at R^3^, however, there was no clear SAR.

### Selectivity and mode of action studies

The selectivity of these series with respect to nearest human homologue of TryR was assessed in glutathione reductase (GR) assays. Both TryR and GR assays were performed with substrate concentrations equal to *K*_m_ allowing direct comparison of IC_50_ values for selectivity assessment. All of the compounds tested demonstrated significant selectivity for TryR over GR (Table 4). This is not surprising if we assume our binding model is correct as the binding site in GR is smaller, less hydrophobic and less negatively charged than that of TryR. Hence, if our compounds are binding by a general hydrophobic interaction and interaction with negatively charged residues in the active site, they are unlikely to interact with GR.

**Table 4:** Selectivity of hit compounds and mode of inhibition.^[a]^

Compd	Mode of inhibiton	Inhibitor constants [μm]		IC_50_ [μm]		EC_50_ [μm]	
		*K*_i_	*K*_i_′	TryR	hGR^[b]^	Parasite^[c]^	MRC-5^[c]^
1	linear mixed	1.6±0.2	3.2±0.3	1.7	>100	10.3	>50
2	NT^[d]^	–	–	1.1	NT	6.0	18.7
3	NT	–	–	1.2	NT	>50	>50
4	linear mixed	3.0±0.2	4.3±0.3	4.2	74.2	25.6	>50
5	linear mixed	2.0±0.1	3.0±0.2	4.7	33.4	18.7	31.1
6	NT	–	–	7.2	NT	6.9	NT
7	NT	–	–	5.1	NT	16.5	NT
10	NT	–	–	5.8	NT	19.2	NT
12	NT	–	–	14.4	NT	32.1	43.7
19	NT	–	–	8.4	NT	14.5	NT
39	linear uncompetitive	4.6±0.3 (T[S]_2_) 29.4±1.7 (NADPH)	–	8.6	>100	1.22	1.62
40	linear uncompetitive	3.0±0.2 (T[S]_2_) 21.5±1.6 (NADPH)	–	12.8	10.0	1.43	3.2
41	NT	–	–	10.2	NT	8.1	NT
45	NT	–	–	13.0	NT	3.4	12.9

[a] MOI: Compounds **1**–**19** are from hit series 1 and **39**–**45** from hit series 2. [b] hGR: human glutathione reductase. [c] Concentration giving 50% growth inhibition after 73 h exposure. [d] NT: not tested.

Mode of inhibition studies were carried out on key representatives of series 1 and 2. The data indicated that neither compound series acted as simple linear competitive inhibitors with respect to T[S]_2_. The mixed inhibition of compounds in series 1 indicates the compounds can either bind to the free enzyme or to the enzyme–substrate complex, implying that the compounds are not binding in an orientation that excludes binding of T[S]_2_. For series 2, the binding appears to be linear and uncompetitive, with compounds binding only to the enzyme–substrate complex. In enzymes such as TryR that obey a bi bi ping pong reaction mechanism, such an inhibition pattern can arise due to an inhibitor competing for the second independent substrate-binding site, NADPH. However, a similar uncompetitive inhibition pattern was observed when the concentration of the second substrate, NADPH, was varied in the presence of a fixed concentration of T[S]_2_. This finding indicates that compounds from series 2 bind at a site distinct from both substrate-binding sites. Since compounds of series2 have some structural similarity to the FAD co-factor of TryR, we propose that disruption of the orientation or displacement of FAD could account for the uncompetitive inhibition pattern. In theory, both series present significant advantages over competitive inhibitors where build up of substrate trypanothione during inhibition in vivo could reduce the efficacy of the inhibitor. Indeed, substrate build-up in the case of uncompetitive inhibition may even drive binding of the inhibitor.

The most potent compounds were also assessed for their ability to affect *T. brucei* and MRC-5 (prototypical mammalian cell line) proliferation in vitro (Table 4). Series 1 compounds showed weak inhibition of parasite growth. Whilst it is expected that cellular activity is likely to be lower than enzyme activity due to factors such as high intracellular substrate concentration, there was no clear correlation between enzyme inhibition and effect on trypanosomes. In the case of series 2, the cellular activity was more potent than would be predicted by the enzyme assay suggesting that these compounds are either selectively concentrated by the parasites or are acting off-target. However, the latter seems more likely, given the lack of selectivity apparent between the trypanosome and MRC-5 read-outs.

## Conclusions

We have reported the identification of two novel compound series active against TryR in vitro from a high-throughput screening campaign. Both hit series were low molecular weight compounds with lead-like properties suitable for a medicinal chemistry optimisation programme. They are structurally very different to other TryR inhibitors reported in the literature and constitute novel chemical lead structures against TryR.

SAR studies were undertaken for both series. For series 1, there was some discernable SAR. Unfortunately, we were unable to significantly increase the potency of the compounds against the enzyme to a level likely to have therapeutic significance. The TryR active site contains both hydrophobic and acidic regions (for interaction with the spermidine moiety); it is likely that what we are observing is hydrophobic interactions between the hydrophobic regions of our inhibitors and the active site and electrostatic interactions between the positive charge on the inhibitors and the negatively charged region of the active site. In order to get a significant increase in potency, it will be necessary to build in some additional specific interactions between the inhibitor and the enzyme. This process would be greatly aided by a co-crystal structure of an inhibitor bound in the active site. Some cellular activity was observed, which implies that the compounds are able to penetrate into cells. However we believe that to get a significant correlation between enzyme and cellular activity will require enzyme inhibitors that are significantly more potent.

Data from the gene knockout studies indicated that it would be necessary to cause >90% loss in activity of TryR for cell death to occur.^3^ Therefore, either a very potent inhibitor (low nanomolar) of the enzyme is required or some kind of irreversible inhibition. Furthermore, the TryR active site is large and relatively solvent exposed, which may make the discovery of low molecular weight, highly potent, competitive inhibitors problematic, especially in the absence of structural information from inhibitors bound into the active site.

In summary, we have identified two novel series of TryR inhibitors from a HTS screen and have carried out SAR studies. Further work is needed to increase the potency of these compounds to a level where the activity is sufficient to achieve significant cellular activity. This would be greatly facilitated by structural information.

## Experimental Section

### Biology

#### Enzyme-based assays

##### Primary screening assay for TryR

Recombinant TryR from *T. brucei* was initially screened for inhibitors in single-point mode. Each test compound (500 nL) was transferred into 384-well clear polystyrene plates at an appropriate stock concentration. Clomipramine and DMSO were added to the appropriate control wells. Then 37.5 μL of reaction mix (3 nm TryR, 6 μm T[S]_2_, 50 μm DTNB, 40 mm Hepes, 1 mm EDTA, pH 7.4) was added to each well and the reaction initiated by addition of 150 μm NADPH (4 μL). Low signal controls received no NADPH. Plates were incubated for 35 min at room temperature and absorbance measured at 405±8 nm using the Envision platereader (Perkin–Elmer).

Initial single-point screening of compounds was conducted at 30 μm; subsequent ten-point screening was conducted between 30 μm and 1 nm or between 100 μm and 3 nm depending on stock concentrations. The final DMSO level was 1% in all cases.

##### Mode of inhibition studies

An assay mixture consisting of TryR, NADPH and DTNB was made up in 40 mm HEPES; 1 mm EDTA, pH 7.4. Assay mixture (180 μL) containing three different concentrations of test compound was added to three rows of a microtitre plate, a fourth row contained only the assay mixture. The test compound concentration ranged up to ∼1×IC_50_ value. T[S]_2_ was serially diluted across a fifth row of the plate to produce a 12-point range from 500 μm to 5.8 μm. The assay was initiated by transferring 20 μL of the T[S]_2_ row to each of the assay rows. The final 200 μL assay contained 150 μm NADPH; 50 μm DTNB and 20 mUmL^−1^ U TryR.

Reaction rates were measured from the linear increase in absorbance at 412 nm using a Molecular Devices Thermomax plate reader. Individual data sets were plotted as 1/v versus 1/[S] using Grafit 5.0 (Erithacus software) and inspected for diagnostic inhibition patterns. Data sets were then globally fitted by nonlinear regression to the appropriate equation for competitive, mixed or uncompetitive inhibition.

#### Cell-based assays

Measurement of the ability of the compounds to inhibit trypanosome (bloodstream form *T. brucei brucei* single marker) and human (MRC5, human lung fibroblast cells) cell growth was performed using a modification of the cell viability assay previously described by Raz et al.^15^

##### Trypanosome growth assay

Cell culture plates were stamped with 1μL of an appropriate stock concentration of test compound, followed by addition of 200 μL trypanosome culture (HMI9-T,^16^ 10% FCS) to each well at a density of 2000 cells per well (except column 12 receiving media only).

##### MRC-5 growth assay

MRC-5 cells (2000 cells per well) were seeded in 200 μL DMEM, 10% FCS and allowed to adhere overnight. One microlitre of test compound was added to each well on the day of assay initiation.

Trypanosome and MRC-5 plates were incubated at 37°C in an atmosphere of 5% CO_2_ for 69 h, prior to the addition of 20 μL rezasurin (final concentration 50 μm). After a further 4 h, fluorescence was measured (excitation 528 nm; emission 590 nm) using a BioTek flx800 plate reader.

For cell assays, ten-point screening was conducted between 15 μm and 0.5 nm or between 50 μm and 2 nm, depending on stock concentrations. The final DMSO level was 0.5% in all cases.

### Chemistry

^1^H NMR were recorded on either a Bruker Avance DPX 500 or on a Bruker Avance 300 spectrometer. Chemical shifts (δ) are expressed in ppm. Signal splitting patterns are described as singlet (s), doublet (d), triplet (t), quartet (q), multiplet (m), broad (br), or combination thereof. Low resolution electrospray (ES) mass spectra were recorded on a Bruker MicroTof mass spectrometer, run in positive ion mode, using either CH_3_OH, CH_3_OH/H_2_O (95:5) or H_2_O/CH_3_CN (1:1) + 0.1% formic acid as the mobile phase. High resolution electrospray measurements were performed on a Bruker MicroTof mass spectrometer. LC-MS analysis was performed using an Agilent HPLC 1100 with a diode array detector in parallel with a Bruker MicroTof mass spectrometer. A Phenomenex Gemini C18 column 110 A 50×3.0 mm, 5 μm particle size was used. The mobile phase was H_2_O/CH_3_CN + 0.1% HCOOH (80:20→5:95 over 3.5 min, 5:95 for 1.5 min; flow rate 0.5 mLmin^−1^).

Thin layer chromatography (TLC) was carried out on Merck silica gel 60 F254 plates using UV light and/or KMnO_4_ for visualisation. Column chromatography was performed using RediSep® 4 or 12 g silica prepacked columns with the specified eluent system.

Compounds **6**, **7**, **13**, **16**, **17**, **19**, **21**, **23** and **24** were purchased from ChemBridge and compounds **18**, **39**, **40**, **41**, **44**, **45**, **46**, **47** and **49** were purchased from ASINEX and submitted to the screening assay. They were checked for purity by LCMS and were found to be >90% pure.

All reactions were carried out under dry and inert conditions unless otherwise stated.

**6-Bromo-*N*-(2-(dimethylamino)ethyl)-2-(5-methylfuran-2-yl)quinoline-4-carboxamide (1)**: Compound **27** (200 mg, 0.6 mm) and carbonyldiimidazole (CDI; 108 mg, 0.66 μm) were stirred in CH_2_Cl_2_ (15 mL) at 35°C for 20 min. *N*,*N*-dimethylethane-1,2-diamine (58 mg, 0.66 mm) was added, and the solution stirred at RT for 18 h. The solution was diluted with CH_2_Cl_2_ (30 mL) and washed with aq NaHCO_3_ (2×20 mL), H_2_O (1×30 mL) and brine (2×20 mL). The organic layer was dried (MgSO_4_), filtered and concentrated to afford a yellow/brown solid. The crude product was purified by flash column chromatography (6%, CH_3_OH/CH_2_Cl_2_) to give **1** as pale yellow solid (65 mg, 27%): ^1^H NMR (500 MHz, [D_6_]DMSO): *δ*=2.28 (6H, br s, 2CH_3_), 2.44 (3H, s, CH_3_), 2.51 (2H, m, CH_2_), 3.46 (2H, q, *J*=6.3 Hz, CH_2_), 6.39 (1H, m, ArH), 7.38 (1H, d, *J*=3.2 Hz, ArH), 7.91 (1H, d, *J*=2.2 Hz, ArH), 7.92 (1H, m, ArH), 7.96 (1H, m, ArH), 8.4 (1H, d, *J*=2.2 Hz, ArH), 8.85 ppm (1H, br s, NH); LCMS (ES+): *m*/*z* (%): 402 (100) [*M*+H]^+^ (^79^Br), 404 (100) [*M*+H]^+^ (^81^Br); *t*_R_=5.0 min.

**6-Bromo-*N*-(2-(methylamino)ethyl)-2-(5-methylfuran-2-yl)quinoline-4-carboxamide (2)**: Prepared as for **1** from **27** (150 mg, 0.45 mm) and CDI (82 mg, 0.5 mm) in CH_2_Cl_2_ (15 mL). The mixture was stirred at 35°C for 20 min, then treated with *N*-methylethane-1,2-diamine (37 mg, 0.5 mm) and stirred at RT for 18 h. The resultant solid product was collected by filtration and further purified by trituration with hot Et_2_O (3×10 mL) to give **2** as a pale yellow solid (37 mg, 21%): ^1^H NMR (500 MHz, [D_6_]DMSO): *δ*=2.29 (3H, s, CH_3_), 2.40 (3H, s, CH_3_), 2.72 (2H, m, CH_2_), 3.41 (2H, m, CH_2_), 6.39 (1H, br s, ArH), 7.37 (1H, s, ArH), 7.89 (1H, dd *J*=9.0 and *2.2* Hz, ArH), 7.96 (2H, m, 2ArH), 8.34 (1H, d, *J*=2.1 Hz, ArH), 8.88 ppm (1H, br s, NH); LCMS (ES+): *m*/*z* (%): 388 (100) [*M*+H]^+^ (^79^Br), 390 (100) [*M*+H]^+^ (^81^Br); *t*_R_=4.2 min.

**6-Bromo-*N***,***N***,***N*****-trimethyl-2-(2-(5-methylfuran-2-yl)quinoline-4-carboxamido)ethanaminium iodide (3)**: CH_3_I (31 mg, 0.22 mm) was added to a solution of **1** (60 mg, 0.15 mm) in dry acetone (5 mL). The solution was stirred at RT for 48 h then concentrated. A solid was precipitated from the resulting gum on the addition of acetone (15 mL), which was isolated by filtration and washed with acetone (3×10 mL) to afford **3** as a beige solid (50 mg, 61%): ^1^H NMR (500 MHz, [D_6_]DMSO): *δ*=2.41 (3H, s, 2CH_3_), 3.19 (9H, s, 3CH_3_), 3.6 (2H, m, CH_2_), 3.79 (2H, m, CH_2_), 6.39 (1H, br s, ArH), 7.36 (1H, m, ArH), 7.94 (1H, dd, *J*=9.0, 2.2 Hz, ArH), 7.98 (2H, m, 2ArH), 8.34 (1H, d, *J*=2.0 Hz, ArH), 9.25 ppm (1H, br s, NH); LCMS (ES+): *m*/*z* (%): 418 (100) [*M*+H]^+^ (^79^Br), 420 (100) [*M*+H]^+^ (^81^Br); *t*_R_=4.4 min.

**6-Bromo-2-(5-methylfuran-2-yl)-*N*-(2-morpholinoethyl)quinoline-4-carboxamide (4)**: Prepared as for **1** from **27** (150 mg, 0.45 mm), CH_2_Cl_2_ (15 mL), CDI (82 mg, 0.5 mm) and 2-morpholinoethanamine (65 mg, 0.5 mm). The reaction was stirred for 18 h then concentrated. The crude solid was recrystallised from refluxing EtOAc to give **4** as a white crystalline solid (95 mg, 48%): ^1^H NMR (500 MHz, [D_6_]DMSO): *δ*=2.38 (3H, s, CH_3_), 2.48 (2H, m, CH_2_), 2.49 (4H, m, 2CH_2_), 4.49 (2H, m, CH_2_), 4.66 (4H, m, 2CH_2_), 6.39 (1H, m, ArH), 7.38 (1H, d, *J*=3.2 Hz, ArH), 7.91 (1H, d, *J*=2.2 Hz, ArH), 7.92 (1H, m, ArH), 7.96 (1H, m, ArH), 8.32 (1H, d, *J*=2.0 Hz, ArH), 8.86 ppm (1H, br s, NH); LCMS (ES+): *m*/*z* (%): 444 (100) [*M*+H]^+^ (^79^Br), 446 (100) [*M*+H]^+^ (^81^Br); *t*_R_=4.5 min.

**6-Bromo-2-(5-methylfuran-2-yl)-*N*-(pyridin-2-ylmethyl)quinoline-4-carboxamide (5)**: Prepared as for **1** from **27** (150 mg, 0.45 mm), CDI (82 mg, 0.5 mm) and pyrin-2-ylmethanamine (55 mg, 0.5 mm). The reaction was stirred for 18 h then concentrated. The crude solid was purified by trituration with hot Et_2_O (3×10 mL) to give **5** as pale yellow solid (70 mg, 37%): ^1^H NMR (500 MHz, [D_6_]DMSO): *δ*=2.39 (3H, s, CH_3_), 4.79 (2H, m, CH_2_), 6.39 (1H, br s, ArH), 7.35 (1H, dd, *J*=6.7, 5.0 Hz, ArH), 7.41 (1H, d, *J*=3.3 Hz, ArH), 7.49 (1H, d, *J*=7.8 Hz, ArH), 7.84 (1H, dt, *J*=7.7, 1.8 Hz, ArH), 7.92 (1H, dd, *J*=9.0, 2.2 Hz, ArH), 7.99 (1H, d, *J*=9.0 Hz, ArH), 8.03 (1H, s, ArH), 8.52 (1H, s, ArH), 8.61 (1H, m, ArH), 9.51 ppm (1H, br s, NH); LCMS (ES+): *m*/*z* (%): 422 (100) [*M*+H]^+^ (^79^Br), 424 (100) [*M*+H]^+^ (^81^Br); *t*_R_=4.7 min.

**6-Chloro-*N*-(2-(dimethylamino)ethyl)-2-(5-methylfuran-2-yl)quinoline-4-carboxamide (8)**: Compound **29** (468 mg, 1.63 mm) and CDI (260 mg, 1.8 mm) were stirred in CH_2_Cl_2_ (15 mL) for 10 min at RT. *N*,*N*-Dimethylethane-1,2-diamine (158 mg, 1.8 mm) was added, and the resulting solution was stirred at RT for 18 h. The reaction was diluted with CH_2_Cl_2_ (20 mL) and washed with aq NaHCO_3_ (2×20 mL), H_2_O (2×30 mL) and brine (1×50 mL). The organic layer was dried (MgSO_4_), filtered and concentrated in vacuo. Purification by flash column chromatography (2%, CH_3_OH/CH_2_Cl_2_) gave **8** as a white solid (200 mg, 34%): ^1^H NMR (500 MHz, [D_6_]DMSO): *δ*=2.26 (6H, s, 2CH_3_), 2.41 (3H, s, CH_3_), 2.48 (2H, m, CH_2_), 3.47 (2H, m, CH_2_), 6.39 (1H, br s, ArH), 7.34 (1H, br s, ArH), 7.77 (1H, m, ArH), 7.90 (1H, br s, ArH), 7.96 (1H, dd, *J*=9.0, 2.2 Hz, ArH), 8.1 (1H, br s, ArH), 8.84 ppm (1H, br s, NH); LCMS (ES+): *m*/*z* (%): 358 [*M*+H]^+^; *t*_R_=5.0 min.

**7-Bromo-*N*-(2-(dimethylamino)ethyl)-2-(5-methylfuran-2-yl)quinoline-4-carboxamide (9)**: Prepared as for **1** from **31** (265 mg, 0.8 mm), CH_2_Cl_2_ (15 mL), CDI (144 mg, 0.88 mm) and *N*,*N*-dimethylethane-1,2-diamine (77 mg, 0.66 mm). The reaction was stirred at RT for 18 h. Purification by flash column chromatography (3%, CH_3_OH/CH_2_Cl_2_) gave **9** as a white solid (85 mg, 38%): ^1^H NMR (300 MHz, CDCl_3_): *δ*=2.3 (6H, s, 2CH_3_), 2.45 (3H, s, CH_3_), 2.68 (2H, t, *J*=5.8 Hz, CH_2_), 3.70 (2H, q, *J*=5.4 Hz, CH_2_), 6.21 (1H, m, ArH), 7.35 (2H, m, ArH), 7.92 (1H, s, ArH), 8.05 (1H, m, ArH), 8.16 ppm (1H, m, ArH); LCMS (ES+): *m*/*z* (%): 402 (100) [*M*+H]^+^ (^79^Br), 404 (100) [*M*+H]^+^ (^81^Br); *t*_R_=5.1 min.

**8-Bromo-*N*-(2-(dimethylamino)ethyl)-2-(5-methylfuran-2-yl)quinoline-4-carboxamide (10)**: Prepared as for **1** from **32** (200 mg, 0.6 mm), CH_2_Cl_2_ (10 mL), CDI (108 mg, 0.66 mm) and *N*,*N*-dimethylethane-1,2-diamine (58 mg, 0.66 mm). The reaction was stirred for 18 h at RT. Purification by flash column chromatography (3%, CH_3_OH/CH_2_Cl_2_) gave **10** as a white solid (60 mg, 25%): ^1^H NMR (500 MHz, [D_6_]DMSO): *δ*=2.24 (6H, s, 2CH_3_), 2.43 (3H, s, CH_3_), 2.8 (2H, m, CH_2_), 3.46 (2H, m, CH_2_), 6.40 (1H, br s, ArH), 7.38 (1H, d, *J*=3.3 Hz, ArH), 7.47 (1H, m, ArH), 7.91 (1H, s, ArH), 8.12 (1H, dd, *J*=8.4, 1.2 Hz, ArH), 8.17 (1H, dd, *J*=7.5, 1.2 Hz, ArH), 8.84 ppm (1H, br t, NH); LCMS (ES+): *m*/*z* (%): 402 (100) [*M*+H]^+^ (^79^Br), 404 (100) [*M*+H]^+^ (^81^Br); *t*_R_=5.2 min.

**2-(5-Methylfuran-2-yl)-*N*-(pyridin-2-ylmethyl)quinoline-4-carboxamide (11)**: Compound **34** (100 mg, 0.4 mm) and CDI (72 mg, 0.44 mm) were dissolved in CH_2_Cl_2_ (4 mL). The solution was stirred for 10 min at RT, then pyrin-2-ylmethanamine (48 mg, 0.44 mm) was added. The reaction was stirred at RT for 48 h and worked up as previously described (see details for **8**). The product was recrystallised from from Et_2_O to give **11** as a white solid (80 mg, 58%): ^1^H NMR (500 MHz, [D_6_]DMSO): *δ*=2.44 (3H, br s, CH_3_), 4.68 (2H, m, CH_2_), 6.39 (1H, m, ArH), 7.31 (1H, m, ArH), 7.35 (1H, m, ArH), 7.48 (1H, d, *J*=7.8 Hz, ArH), 7.6 (1H, ddd, *J*=8.2, 6.9, 1.2 Hz, ArH), 7.80 (1H, ddd, *J*=8.4, 6.9, 1.4 Hz, ArH), 7.85 (1H, dt, *J*=7.7, 1.8 Hz, ArH), 7.97 (1H, s, ArH), 8.05 (1H, d, *J*=8.0 Hz, ArH), 8.16 (1H, m, ArH), 8.58 (1H, ddd, *J*=4.8, 1.6 and 0.8 Hz, ArH), 9.47 ppm (1H, br s, NH); LCMS (ES+): *m*/*z* (%): 344 [*M*+H]^+^; *t*_R_=4.7 min.

***N*****-(2-(Dimethylamino)ethyl)-2-(5-methylfuran-2-yl)quinoline-4-carboxamide (12)**: Prepared as for **11** from **34** (100 mg, 0.4 mm), 8 mL CH_2_Cl_2_, CDI (72 mg, 0.44 mm) and *N*,*N*-dimethylaminoethylenediamine (39 mg, 0.44 mm). The reaction was stirred for 18 h at RT. Column chromatography (5%, CH_3_OH/CH_2_Cl_2_) gave **12** as a white solid (30 mg, 24%): ^1^H NMR (500 MHz, [D_6_]DMSO): *δ*=2.22 (6H, s, 2CH_3_), 2.41 (3H, s, CH_3_), 2.48 (2H, m, CH_2_), 3.46 (2H, m, CH_2_), 6.39 (1H, m, ArH), 7.32 (1H, d, *J*=3.3 Hz, ArH), 7.59 (1H, ddd, *J*=8.2, 6.9, 1.2 Hz, ArH), 7.78 (1H, ddd, *J*=8.4, 6.9, 1.4 Hz, ArH), 7.86 (1H, s, ArH), 8.03 (1H, d, *J*=8.4 Hz, ArH), 8.11 (1H, m, ArH), 8.8 ppm (1H, br s, NH); LCMS (ES+): *m*/*z* (%): 324 [*M*+H]^+^; *t*_R_=4.3 min.

**2-(5-Methylfuran-2-yl)-*N*-(2-morpholinoethyl)quinoline-4-carboxamide (14)**: Prepared as for **11** from **34** (150 mg, 0.6 mm), 10 mL CH_2_Cl_2_, CDI (108 mg, 0.66 mm) and 2-morpholinoethanamine (86 mg, 0.66 mm). The reaction was stirred for 18 h at RT, concentrated and purified by triturating the crude solid with hot Et_2_O to give **14** (50 mg, 23%): ^1^H NMR (500 MHz, [D_6_]DMSO): *δ*=2.41 (3H, s, CH_3_), 2.48 (4H, m, 2CH_2_), 2.52 (2H, m, CH_2_), 3.49 (2H, q, *J*=6.5 Hz, CH_2_), 3.64 (4H, t, *J*=4.4 Hz, 2CH_2_), 6.39 (1H, m, ArH), 7.34 (1H, d, *J*=3.3 Hz, ArH), 7.59 (1H, ddd, *J*=8.2, 6.9, 1.2 Hz, ArH), 7.79 (1H, ddd, *J*=8.4, 6.9, 1.4 Hz, ArH), 7.85 (1H, s, ArH), 8.05 (1H, d, *J*=8.4 Hz, ArH), 8.18 (1H, m, ArH), 8.79 ppm (1H, br s, NH); LCMS (ES+): *m*/*z* (%): 366 [*M*+H]^+^; *t*_R_=4.1 min.

***N*****-Benzyl-6-bromo-2-(5-methylfuran-2-yl)quinoline-4-carboxamide (15)**: Prepared as for **1** from **27** (150 mg, 0.45 mm), CH_2_Cl_2_ (10 mL), CDI (82 mg, 0.5 mm) and benzylamine (54 mg, 0.5 mm). The reaction was stirred for 18 h at RT. Flash column chromatography (2%, CH_3_OH/CH_2_Cl_2_) gave **15** as a beige solid (16 mg, 8%): ^1^H NMR (500 MHz, CDCl_3_): *δ*=2.36 (3H, s, CH_3_), 4.70 (2H, d, *J*=5.7 Hz, CH_2_), 6.09 (1H, m, ArH), 6.59 (1H, br s, NH), 6.95 (1H, m, ArH), 7.31 (1H, m, ArH), 7.39 (4H, m, 4ArH), 7.58 (1H, s, ArH), 7.66 (1H, dd, *J*=9.0, 2.1 Hz, ArH), 7.74 (1H, d, *J*=9.0 Hz, ArH), 8.2 ppm (1H, d, *J*=2.1 Hz, ArH); LCMS (ES+): *m*/*z* (%): 421 (100) [*M*+H]^+^ (^79^Br), 423 (100) [*M*+H]^+^ (^81^Br); *t*_R_=5.7 min.

**6-Bromo-*N*-(2-(dimethylamino)ethyl)-2-(furan-2-yl)quinoline-4-carboxamide (20)**: Prepared as for **1** from **33** (200 mg, 0.63 mm), CH_2_Cl_2_ (15 mL), CDI (115 mg, 0.7 mm) and *N*,*N*-dimethylethane-1,2-diamine (62 mg, 0.7 mm). The reaction was stirred at RT for 18 h. Flash column chromatography (6%, CH_3_OH/CH_2_Cl_2_) gave **20** as a pale yellow solid (65 mg, 27%): ^1^H NMR (500 MHz, [D_6_]DMSO): *δ*=2.29 (6H, br s, 2CH_3_), 2.51 (2H, m, CH_2_), 3.48 (2H, q, *J*=6.5 Hz, CH_2_), 6.78 (1H, m, ArH), 7.47 (1H, m, ArH), 7.95 (1H, dd, *J*=9.0, 2.2 Hz, ArH), 7.99 (3H, m, 3ArH), 8.44 (1H, d, *J*=2.2 Hz, ArH), 8.9 ppm (1H, br s, NH); LCMS (ES+): *m*/*z* (%): 388 (100) [*M*+H]^+^ (^79^Br), 390 (100) [*M*+H]^+^ (^81^Br); *t*_R_=4.9 min.

**6-Bromo-*N*-(2-(dimethylamino)ethyl)-2-(5-methylthiophen-2-yl)quinoline-4-carboxamide (22)**: Prepared as for **11** from **30** (209 mg, 0.6 mm), CH_2_Cl_2_ (15 mL), CDI (108 mg, 0.66 mm) and *N*,*N*-dimethylethane-1,2-diamine (58 mg, 0.66 mm). The reaction was stirred for 18 h at RT. Flash column chromatography (3%, CH_3_OH/CH_2_Cl_2_) gave **22** as a beige solid (73 mg, 29%): ^1^H NMR (500 MHz, [D_6_]DMSO): *δ*=2.28 (6H, br s, 2CH_3_), 2.44 (3H, s, CH_3_), 2.60 (2H, m, CH_2_), 3.50 (2H, q, *J*=6.3 Hz, CH_2_), 6.92 (1H, br s, ArH), 7.76 (1H, m, ArH), 7.89 (2H, m, 2ArH), 8.11 (1H, s, ArH), 8.40 (1H, d, *J*=2.1 Hz, ArH), 8.9 ppm (1H, m, NH); LCMS (ES+): *m*/*z* (%): 419 (100) [*M*+H]^+^ (^79^Br), 421 (100) [*M*+H]^+^ (^81^Br); *t*_R_=5.1 min.

**6-bromo-2-(5-methylfuran-2-yl)-*N*-phenethylquinoline-4-carboxamide (25)**: Prepared as for **1** from **27** (200 mg, 0.6 mm), CH_2_Cl_2_ (10 mL), CDI (108 mg, 0.66 mm) and phenethylamine (84 μL, 0.66 mm). The reaction was stirred for 18 h at RT. Purification by flash column chromatography (3%, CH_3_OH/CH_2_Cl_2_) gave **25** as a cream solid (58 mg, 28%): ^1^H NMR (500 MHz, CDCl_3_): *δ*=2.43 (3H, s, CH_3_), 4.70 (2H, d, *J*=5.7 Hz, CH_2_), 5.23 (2H, m, CH_2_), 6.11 (1H, m, ArH), 6.58 (1H, m, ArH), 6.97 (1H, d, *J*=3.3 Hz, ArH), 7.31 (1H, m, ArH), 7.61 (3H, m, 3ArH), 7.61 (1H, s, ArH), 7.66 (1H, dd, *J*=9.0, 2.1 Hz, ArH), 7.74 (1H, d, *J*=9.0 Hz, ArH), 8.21 ppm (1H, d, *J*=2.1 Hz, ArH); LCMS (ES+): *m*/*z* (%): 434 (100) [*M*+H]^+^ (^79^Br), 436 (100) [*M*+H]^+^ (^81^Br); *t*_R_=5.2 min.

***N*****-(2-(dimethylamino)ethyl)-6-fluoro-2-(5-methylfuran-2-yl)quinoline-4-carboxamide (26)**: Prepared as for **1** from **28** (150 mg, 0.55 mm), CH_2_Cl_2_ (10 mL), CDI (90 mg, 0.61 mm) and *N*,*N*-dimethylethane-1,2-diamine (54 mg, 0.61 mm). The reaction was stirred for 18 h at RT. Purification by flash column chromatography (3%, CH_3_OH/CH_2_Cl_2_) gave **26** as a white solid (50 mg, 24%): ^1^H NMR (500 MHz, [D_6_]DMSO): *δ*=2.29 (6H, br s, 2CH_3_), 2.46 (3H, s, CH_3_), 2.53 (2H, m, CH_2_), 3.46 (2H, q, *J*=6.5 Hz, CH_2_), 6.37 (1H, dd, *J*=3.3, 1.0 Hz, ArH), 7.34 (1H, d, *J*=3.3 Hz, ArH), 7.72 (1H, m, ArH), 7.92 (1H, br s, ArH), 7.95 (1H, dd, *J*=10.4, 2.9 Hz, ArH), 8.1 (1H, dd, *J*=9.3, 5.6 Hz, ArH), 8.85 ppm (1H, m, NH); LCMS (ES+): *m*/*z* (%): 341 [*M*+H]^+^; *t*_R_=4.0 min.

**6-Bromo-2-(5-methylfuran-2-yl)quinoline-4-carboxylic acid (27)**: A solution of 5-bromoindoline-2,3-dione (1.25 g, 5 mm) in EtOH/H_2_O (20 mL, 1:1) in a 30 mL microwave reactor tube was treated with 1-(5-methylfuran-2-yl)ethanone (0.62 g, 5 mm). This tube was transferred to the microwave reactor and, following a 1 min pre-stirring, was heated to 110°C for 13 min. The reaction mixture was acidified to pH 3 using AcOH, diluted with H_2_O (60 mL) and extracted with EtOAc (2×80 mL). The organic layer was washed with H_2_O (2×80 mL) and brine (80 mL), dried (MgSO_4_), filtered and concentrated to give a yellow/brown solid. The crude product was redissolved in a minimum amount of hot EtOAc (∼40 mL) and the pure product was allowed to crystallise out yielding **27** (500 mg, 30%): *R*_F_=0.1 (10%, CH_3_OH/CH_2_Cl_2_); ^1^H NMR (500 MHz, [D_6_]DMSO): *δ*=2.42 (3H, br s, CH_3_), 6.40 (1H, dd, *J*=3.3, 0.9 Hz, ArH), 7.39 (1H, d, *J*=3.3 Hz, ArH), 7.83 (1H, dd, *J*=9.0, 2.4 Hz, ArH), 8.07 (1H, d, *J*=9.0 Hz, ArH), 8.31 (1H, s, ArH), 8.77 (1H, d, *J*=2.4 Hz, ArH), 14.16 ppm (1H, br s, COOH); LCMS (ES+): *m*/*z* (%): 332 (100) [*M*+H]^+^ (^79^Br), 334 (100) [*M*+H]^+^ (^81^Br); *t*_R_=0.5–0.6 min.

**6-Fluoro-2-(5-methylfuran-2-yl)quinoline-4-carboxylic acid (28)**: Prepared as for **27** from 5-fluoroindoline-2,3-dione (0.94 g, 5.51 mm), 1-(5-methylfuran-2-yl)ethanone (0.65 mL, 6.06 mm) and KOH (2.8 g, 50 mm) in EtOH/H_2_O (20 mL, 1:1) to give **28** as a pale cream solid (600 mg, 40%): ^1^H NMR (500 MHz, [D_6_]DMSO): *δ*=2.45 (3H, br s, CH_3_), 6.40 (1H, m, ArH), 7.40 (1H, d, *J*=3.3 Hz, ArH), 7.94 (1H, dd, *J*=9.0, 2.2 Hz, ArH), 8.00 (1H, d, *J*=8.9 Hz, ArH), 8.31 (1H, s, ArH), 8.92 (1H, d, *J*=2.1 Hz, ArH), 14.10 ppm (1H, br s, COOH); LCMS (ES+): *m*/*z* (%): 271 [*M*+H]^+^; *t*_R_=0.5–0.6 min.

**6-Chloro-2-(5-methylfuran-2-yl)quinoline-4-carboxylic acid (29)**: Prepared as for **27** from 5-chloroindoline-2,3-dione (1 g, 5.51 mm), 1-(5-methylfuran-2-yl)ethanone (0.65 mL, 6.06 mm) and KOH (2.8 g, 50 mm) in EtOH/H_2_O (20 mL, 1:1) to give **29** as a pale cream solid (600 mg, 38%): ^1^H NMR and LCMS confirm purity at >95%; ^1^H NMR (500 MHz, [D_6_]DMSO): *δ*=2.42 (3H, br s, CH_3_), 6.40 (1H, br d, *J*=3.2 Hz, ArH), 7.39 (1H, d, *J*=3.3 Hz, ArH), 7.94 (1H, dd, *J*=2.2, 9.0 Hz, ArH), 8.00 (1H, d, *J*=8.9 Hz, ArH), 8.3 (1H, s, ArH), 8.92 (1H, m, ArH), 14.15 ppm (1H, br s, COOH); LCMS (ES+): *m*/*z* (%): 288 [*M*+H]^+^; *t*_R_=0.5–0.6 min.

**6-Bromo-2-(5-methylthiophen-2-yl)quinoline-4-carboxylic acid (30)**: Prepared as for **27** from 2-acetyl-5-methylthiophene (0.77 g, 5.5 mm) to give carboxylic acid **30** as a yellow solid (1.08 g, 62%): ^1^H NMR and LCMS confirm purity at >95%; ^1^H NMR (500 MHz, [D_6_]DMSO): *δ*=2.47 (3H, br s, CH_3_), 6.92 (1H, m, ArH), 7.92 (3H, m, 3ArH), 8.42 (1H, s, ArH), 8.88 (1H, br s, ArH), 14.15 ppm (1H, br s, COOH); LCMS (ES+): *m*/*z* (%): 348 (100) [*M*+H]^+^ (^79^Br), 350 (100) [*M*+H]^+^ (^81^Br); *t*_R_=0.5–0.6 min.

**7-Bromo-2-(5-methylfuran-2-yl)quinoline-4-carboxylic acid (31)**: Prepared as for **27** from 6-bromoindoline-2,3-dione (1 g, 3.3 mm). The crude product was triturated with hot Et_2_O to isolate purified carboxylic acid **31** as a pale brown solid (350 mg, 36%): ^1^H NMR and LCMS confirm purity at >95%; ^1^H NMR (500 MHz, [D_6_]DMSO): *δ*=2.42 (3H, s, CH_3_), 6.40 (1H, m, ArH), 7.42 (1H, m, ArH), 7.50 (1H, m, ArH), 8.18 (1H, s, ArH), 8.22 (1H, s, ArH), 8.54 (1H, m, ArH), 14.2 ppm (1H, br s, COOH); LCMS (ES+): *m*/*z* (%): 332 (100) [*M*+H]^+^ (^79^Br), 334 (100) [*M*+H]^+^ (^81^Br); *t*_R_=0.5–0.6 min.

**8-Bromo-2-(5-methylfuran-2-yl)quinoline-4-carboxylic acid (32)**: Prepared as for **27** from 7-bromoindoline-2,3-dione (1 g, 3.3 mm). The crude product was triturated with hot Et_2_O to isolate purified carboxylic acid **32** as a pale brown solid (400 mg, 41%): ^1^H NMR and LCMS confirm purity at >95%; ^1^H NMR (500 MHz, [D_6_]DMSO): *δ*=2.42 (3H, s, CH_3_), 6.42 (1H, m, ArH), 7.39 (1H, d, *J*=3.3 Hz, ArH), 7.53 (1H, dd, *J*=8.4, 7.6 Hz, ArH), 8.2 (1H, dd, *J*=7.5, 1.1 Hz, ArH), 8.28 (1H, s, ArH), 8.62 (1H, dd, *J*=8.5, 1.1 Hz, ArH), 14.2 ppm (1H, br s, COOH); LCMS (ES+): *m*/*z* (%): 332 (100) [*M*+H]^+^ (^79^Br), 334 (100) [*M*+H]^+^ (^81^Br); *t*_R_=0.5–0.6 min.

**6-Bromo-2-(furan-2-yl)quinoline-4-carboxylic acid (33)**: 5-Bromoindoline-2,3-dione (1.25 g, 5 mm) and KOH flakes (2.8 g, 50 mm) were added to a 30 mL microwave reactor tube containing EtOH/H_2_O (20 mL, 1:1). The tube was transferred to the microwave reactor and, following a 1 min prestirring, was heated to 110°C for 3 min. The tube was then removed from the reactor and 2-acetylfuran (0.6 g, 5.5 mm) was added. Heating in the microwave was recommenced (110°C for 12 min) to achieve complete reaction. The reaction mixture was then acidified to pH 3 using AcOH, diluted with H_2_O (60 mL) and extracted with EtOAc (2×80 mL). The organic layer was washed with H_2_O (2×80 mL) and brine (80 mL), dried (MgSO_4_), filtered and concentrated to give a pale yellow solid. The crude solid was heated to reflux in EtOAc (20 mL) and the remaining insoluble cream solid was filtered off to give **33** (900 mg, 60%): Product purity was determined by ^1^H NMR and LCMS as >98%; *R*_F_=0.3 (10%, CH_3_OH/CH_2_Cl_2_); ^1^H NMR (500 MHz, [D_6_]DMSO): *δ*=6.77 (1H, m, ArH), 7.50 (1H, d, *J*=3.4 Hz, ArH), 7.97 (1H, dd, *J*=9.0, 2.1 Hz, ArH), 8.01 (2H, m, 2ArH), 8.38 (1H, s, ArH), 8.91 (1H, m, ArH), 14.17 ppm (1H, br s, COOH); LCMS (ES+): *m*/*z* (%): 318 [*M*+H]^+^; *t*_R_=0.5–0.6 min.

**2-(5-Methylfuran-2-yl)quinoline-4-carboxylic acid (34)**: Prepared as for **23** from indoline-2,3-dione (1 g, 6.79 mm), KOH flakes (3.8 g, 50 mm) and 2-acetyl-5-methylfuran (0.79 mL, 6.79 mm). The crude solid was redissolved in refluxing EtOAc (30 mL) and the purified product was precipitated out and filtered off, washing with cold EtOAc (20 mL) to give compound **34** (500 mg, 31%): Product purity was determined by ^1^H NMR and LCMS as >98%; *R*_F_=0.15 (10%, CH_3_OH/CH_2_Cl_2_); ^1^H NMR (500 MHz, [D_6_]DMSO): *δ*=2.42 (3H, br s, CH_3_), 6.38 (1H, m, ArH), 7.36 (1H, d, *J*=3.3 Hz, ArH), 7.62 (1H, ddd, *J*=8.2, 6.9, 1.2 Hz, ArH), 7.79 (1H, ddd, *J*=8.4, 6.9, 1.4 Hz, ArH), 7.87 (1H, s, ArH), 8.02 (1H, d, *J*=8.4 Hz, ArH), 8.11 ppm (1H, m, ArH); LCMS (ES+): *m*/*z* (%): 254 [*M*+H]^+^; *t*_R_=0.5–0.6 min.

**1-Methyl-6-(1-methylhydrazinyl)pyrimidine-2,4-(1*H*,3*H*)dione (35)**: 6-Chloro-3-methyluracil (2.0 g, 12.5 mmol) and methyl-hydrazine (0.861 mL, 16.2 mmol) were refluxed in EtOH (20 mL) for 1.5 h. The reaction was cooled to RT and concentrated in vacuo to afford pyrimidine **35** as a pale yellow solid (1.96 g, 100%): ^1^H NMR (500 MHz, [D_6_]DMSO): *δ*=2.67 (1H, br s, NH), 3.09 (3H, s, CH_3_), 3.11 (3H, s, CH_3_), 3.41 (2H, br s, NH_2_), 4.23 ppm (1H, s, CH); LCMS (ES+): *m*/*z* (%): 288 [*M*+H]^+^ (100); *t*_R_=2.7–2.9 min.

**6-(2-(5-Bromo-2-fluorobenzylidene)-1-methylhydrazinyl)-1-methylpyrimidine-2,4(1*H*,3*H*)dione (36)**: Compound **35** (1.0 g, 4.07 mmol) and 5-bromo-2-fluoro-benzaldehyde (0.959 mL, 4.07 mmol) were refluxed in EtOH (40 mL) for 2 h. The reaction was cooled to RT, filtered and washed with Et_2_O to afford benzylidene **36** as a cream solid (1.61 g, 100%): ^1^H NMR (500 MHz, [D_6_]DMSO): *δ*=3.13 (2H, s, CH_3_), 3.39 (3H, s, CH_3_), 5.31 (1H, d, *J*=2.3 Hz, CH), 7.28 (1H, dd, *J*=10.4, 8.8 Hz, ArH), 7.60 (1H, ddd, *J*=8.8, 4.6, 2.6 Hz, ArH), 7.88 (1H, s, NH), 8.74 (1H, dd, *J*=6.6, 2.6 Hz, ArH), 11.05 ppm (1H, d, *J*=2.3 Hz, imine CH); LCMS (ES+): *m*/*z* (%): 381 (100) [*M*+H]^+^ (^79^Br), 383 (100) [*M*+H]^+^ (^81^Br); *t*_R_=3.9 min.

**3-(4-Bromo-2-fluorophenyl)-4-hydroxy-8-methyl-6,8-dihydropyrimido[5,4-e,1,2,4]triazine-5,7(1*H*,4*H*)dione (37)**: Benzylidene **36** (0.443 g, 1.22 mmol) was dissolved in AcOH/H_2_0 (3.45/0.17 mL) and the solution was cooled to 0°C. NaNO_2_ (0.126 g, 1.83 mmol) was added portionwise and the reaction was warmed to RT over 16 h. Et_2_O (10 mL) was added and the reaction was stirred at RT for 1 h. The resultant yellow precipitate was filtered and washed with Et_2_O to afford **37** (0.460 g, 99%): ^1^H NMR (500 MHz, [D_6_]DMSO): *δ*=3.25 (3H, s, CH_3_), 7.39 (1H, dd, *J*=10.6, 8.8 Hz, ArH), 7.72 (1H, ddd, *J*=8.8, 4.1, 2.7 Hz, ArH), 8.11 (1H, dd, *J*=6.8, 2.7 Hz, ArH), 12.30 ppm (1H, br s, OH).

**3-(5-Bromo-2-fluorophenyl)-6-methylpyrimido[5,4-*e*,1,2,4]triazine-5,7(1*H*,6*H*)dione (42)**: Compound **37** (0.430 g, 1.12 mmol) was heated in anhyd DMF (5 mL) at 90°C for 2 h. The reaction was cooled to RT and iced H_2_O was added. The resultant brown precipitate was filtered and dried in vacuo to afford pyrimido triazine **42** (0.357 g, 91%): ^1^H NMR (500 MHz, [D_6_]DMSO): *δ*=3.30 (3H, s, CH_3_), 7.48 (1H, dd, *J*=10.4, 8.9 Hz, ArH), 7.86 (1H, ddd, *J*=8.9, 4.1, 2.7 Hz, ArH), 8.15 (1H, dd, *J*=6.6, 2.7 Hz, ArH) and 13.00 ppm (br s, 1H, NH); LCMS (ES+): *m*/*z* (%): 351 (100) [*M*+H]^+^ (^79^Br), 353 (100) [*M*+H]^+^ (^81^Br); *t*_R_=3.9 min.

**3-(5-Bromo-2-fluorophenyl)pyrimido[5,4-*e*,1,2,4]triazine-5,7(1*H*,6*H*)dione (43)**: 3-(5-Bromo-2-fluorophenyl)-4-hydroxy-1-methyl-6,8-dihydropyrimido[5,4-*e*][1,2,4]triazine-5,7(1*H*,4*H*)-dione (0.430 g, 1.12 mmol) was heated in anhyd DMF (5 mL) at 90°C for 3 h in a sealed tube with 4 Å molecular sieves. The reaction was cooled to RT and iced H_2_O was added. The resultant brown precipitate was filtered and dried in vacuo to afford pyrimido triazine **43** (48 mg, 77%): ^1^H NMR (500 MHz, [D_6_]DMSO): *δ*=7.29 (1H, dd, *J*=5.0, 3.7 Hz, ArH), 7.86 (1H, ddd, *J*=5.0, 1.2 Hz, ArH), 8.01 (1H, dd, *J*=3.7, 1.2 Hz, ArH), 11.97 (1H, br s, NH), 12.49 ppm (1H, br s, NH).

**3-(5-Bromo-2-fluorophenyl)-1-((2-dimethylamino)ethyl)-6-methylpyrimido[5,4-e,1,2,4]triazine-5,7(1*H*,6*H*)dione (48)**: Compound **42** (50 mg, 0.14 mmol), Cs_2_CO_3_ (97 mg, 0.30 mmol), 2-dimethylaminoethylchloride (20 mg, 0.14 mmol) and anhyd DMF (2 mL) were heated at 80°C for 3 h. The reaction mixture was then filtered and diluted with EtOAc, the organic layer was washed with H_2_O (3×10 mL), dried (MgSO_4_), filtered and concentrated in vacuo. The crude was purified by column chromatography (CH_2_Cl_2_/MeOH/NH_3_, 95:5:0.1) to give pyrdimido triazine **48** as a yellow solid (5 mg, 9%): ^1^H NMR (500 MHz, CDCl_3_): *δ*=2.11 (6H, br s, 2CH_3_), 2.80 (2H, br s, CH_2_), 3.48 (3H, s, CH_3_), 4.13 (2H, t, *J*=6.2 Hz, CH_2_), 7.04 (1H, dd, *J*=10.3, 8.8 Hz, ArH), 7.49 (1H, ddd, *J*=8.8, 4.2, 2.6 Hz, ArH,), 8.13 ppm (1H, dd, *J*=6.6, 2.6 Hz, ArH); LCMS (ES+): *m*/*z* (%): 395 (100) [*M*+H]^+^ (^79^Br), 397 (100) [*M*+H]^+^ (^81^Br); *t*_R_=0.9–1.1 min.

**3-(5-Bromo-2-fluorophenyl)-1-isopropyl-6-methylpyrimido[5,4-e,1,2,4]triazine-5,7(1*H*,6*H*)dione (50)**: Compound **42** (50 mg, 0.14 mmol), Cs_2_CO_3_ (97 mg, 0.30 mmol), isopropylbromide (0.015 mL, 0.14 mmol) and anhyd DMF (2 mL) were heated at 150°C for 10 min in a Biotage microwave reactor. The reaction mixture was filtered and the filtrate was concentrated in vacuo. The crude was purified by column chromatography (CH_2_Cl_2_/MeOH/NH_3_, 95:5:0.1) to give pyrdimido triazine **50** as a yellow solid (5 mg, 9%): ^1^H NMR (500 MHz, CDCl_3_): *δ*=8.35 (1H, dd, *J*=6.6, 2.6 Hz, ArH), 7.65 (1H, ddd, *J*=8.8, 4.2, 2.6 Hz, ArH), 7.18 (1H, dd, *J*=10.3, 8.8 Hz, ArH,), 5.90 (1H, sep, *J*=7.0 Hz, CH), 3.57(3H, s, CH_3_), 1.72 ppm (6H, d, *J*=7.0 Hz, isopropy-CH_3_); LCMS (ES+): *m*/*z* (%): 399 (100) [*M*+H]^+^ (^79^Br), 401 (100) [*M*+H]^+^ (^81^Br); *t*_R_=4.8 min.

**3-(5-Bromo-2-fluorophenyl)-1-butyl-6-methylpyrimido[5,4-*e*,1,2,4]triazine-5,7(1*H*,6*H*)dione (51)**: Compound **42** (50 mg, 0.14 mmol), Cs_2_CO_3_ (69 mg, 0.21 mmol), *n*-butylbromide (0.015 mL, 0.14 mmol) and anhyd DMF (2 mL) were heated at 80°C for 6 h. The reaction mixture was filtered and the filtrate was concentrated in vacuo. The crude was purified by column chromatography (hexane/Et_2_O, 9:1) to give pyrdimido triazine **51** as a yellow solid (5 mg, 9%): ^1^H NMR (500 MHz, CDCl_3_): *δ*=8.33 (1H, dd, *J*=6.6, 2.6 Hz, ArH), 7.53 (1H, ddd, *J*=8.7, 4.2, 2.6 Hz, ArH), 7.16 (1H, dd, *J*=10.2, 8.8 Hz, ArH,), 4.53 (2H, t, *J*=7.7 Hz, CH_2_), 3.56 (3H, s, CH_3_), 1.81–1.78 (2H, m, CH_2_), 1.50–1.43 (2H, m, CH_2_) and 1.00–0.97 ppm (3H, m, CH_3_).

**3-(5-Bromo-2-fluorophenyl)-1-cyclopentyl-6-methylpyrimido[5,4-*e*,1,2,4]triazine-5,7(1*H*,6*H*)dione (52)**: Compound **42** (50 mg, 0.14 mmol), K_2_CO_3_ (40 mg, 0.29 mmol), cyclopentylbromide (0.020 mL, 0.14 mmol) and anhyd DMF (2 mL) were heated at 150°C for 10 min in a Biotage microwave reactor. The reaction mixture was filtered and the filtrate concentrated in vacuo. The crude was purified by column chromatography (CH_2_Cl_2_/MeOH/NH_3_ 95:5:0.1) to give pyrdimido triazine **52** as a yellow solid (5 mg, 9%): ^1^H NMR (500 MHz, CDCl_3_): *δ*=8.19 (1H, dd, *J*=6.7, 2.6 Hz, ArH), 7.54 (1H, ddd, *J*=8.8, 4.2, 2.6 Hz, ArH), 7.11 (1H, dd, *J*=10.4, 8.8 Hz, ArH,), 4.96 (1H, m, cyclopentyl CH), 3.51 (3H, s, CH_3_), 2.36–2.31 (2H, m, cyclopentyl CH_2_), 2.09–2.03 (4H, m, cyclopentyl CH_2_) and 1.73–1.65 ppm (2H, m, cyclopentyl CH_2_).
