# Supplementary material for: Investigation of Trypanothione Reductase as a Drug Target in Trypanosoma brucei
Source: ChemMedChem. 2009 Nov 18;4(12):2060–9. doi: 10.1002/cmdc.200900262 (PMC2855869; doi:10.1002/cmdc.200900262)
Supplement: Supplementary file 1 [file cmdc0004-2060-SD1.pdf]

CHEMMEDCHEM
Chemistry & Drug Discovery

Supporting Information

© Copyright Wiley-VCH Verlag GmbH & Co. KGaA, 69451 Weinheim, 2009

Supporting Information

Investigation of Trypanothione Reductase as a Drug Target in *Trypanosoma brucei*

Daniel Spinks[¥], Emma J. Shanks[¥], Laura A. T. Cleghorn[¥], Stuart McElroy, Deuan Jones, Daniel James, Alan H. Fairlamb, Julie A. Frearson, Paul G. Wyatt and Ian H. Gilbert*

[¥] these authors contributed equally to the study

Drug Discovery Unit, College of Life Sciences, University of Dundee, Sir James Black Centre, Dundee, DD1 5EH, UK
* e-mail: i.h.gilbert@dundee.ac.uk; fax: +44 1382 386 373

Quality parameters in hit discovery campaign for TryR

The initial TryR screening campaign was conducted in single point, where each of 61,808 compounds was tested at 30 µM,and percent inhihbition (PI) calculated. The performance statistics for the screen are shown in Figure S1.

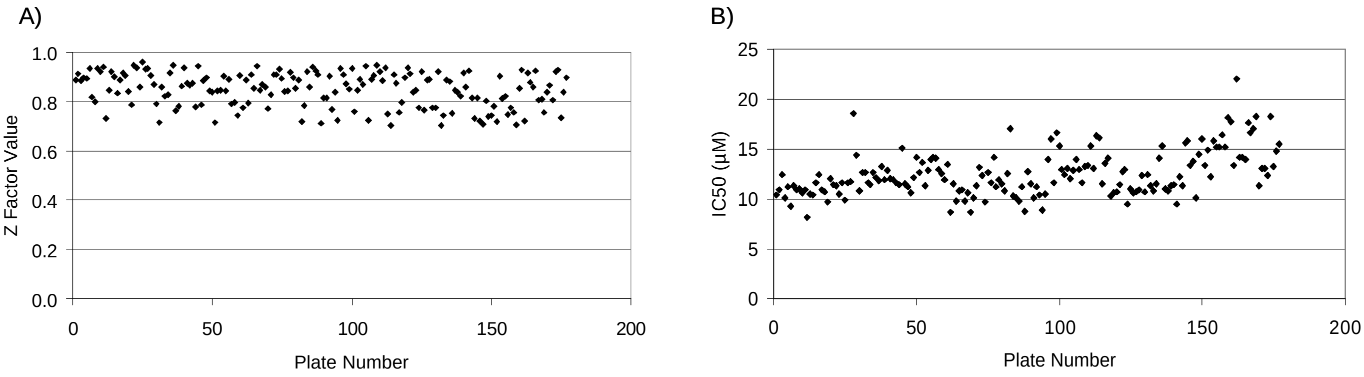

Figure S1. Data quality parameters throughout screening campaign A) Z factor and B) IC_{50} of clomipramine (standard inhibitor included on all screening plates, n = 177)

Based on a statistical analysis of the error around the full signal controls (5xSD), compounds with PI values ≥ 50% (n = 901) were cherry-picked for retest. Additionally, compounds with PI < 30 (n = 16) were also re-tested to ensure they were not automation errors creating false negatives. PI was then determined at 30 µM in duplicate, as described for the single point screen. The correlation between replicates within the retest screen and correlation with primary screen was very good (Figure S2).

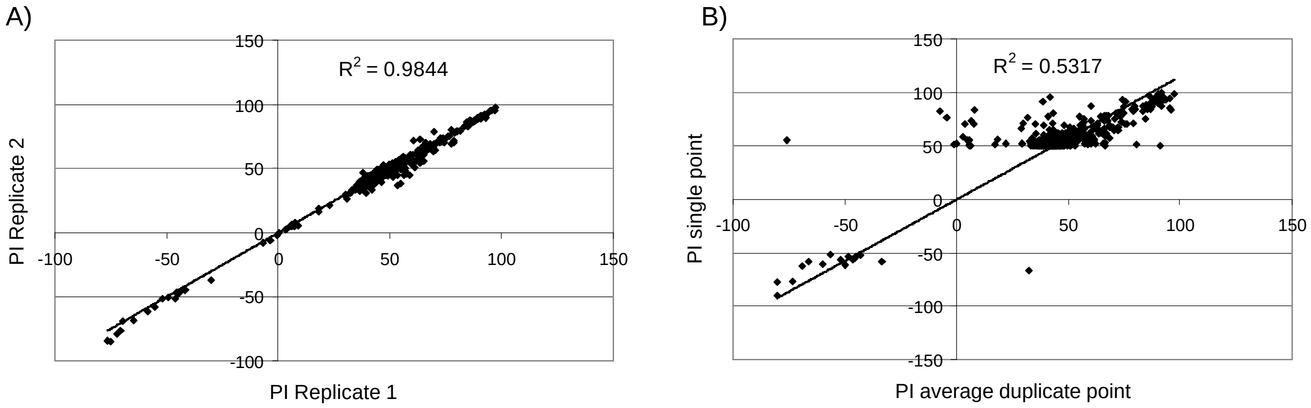

Figure S2. Correlation plots showing A) PI values obtained from individual replicate points in the retest screen and PI values obtained from the single point screen and the average from the retest screen replicates

Potency Screening

All potency curves were tested in duplicate. Two titrations of the standard inhibitor clomipramine were included on all potency plates, as a measure of consistency between assays. All screening plates were approved on the basis of attaining certain quality thresholds (Z'>0.6; IC_{50} clomipramine between 6 – 24 µM).

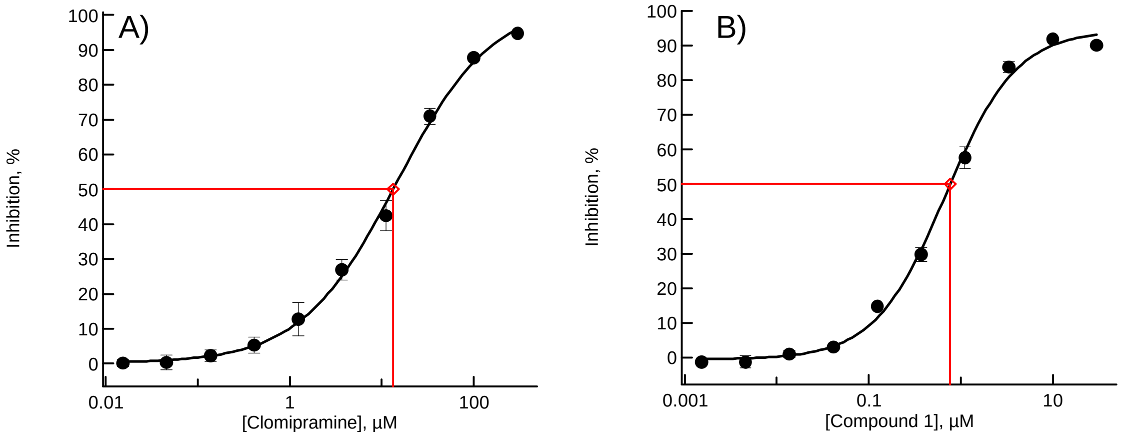

Figure S3. Example potency curves. Concentration-dependent inhibition by A) clomipramine (n = 10). IC_{50} value was determined as 13.31 ± 1.65 µM (Hill Slope = 0.85 ± 0.1) and B) Compound 1 (n = 2), identified as a hit compound against TryR. IC_{50} value was determined as 0.77 ± 0.048 µM (Hill Slope = 1.13 ± 0.05). (Mean ± SD)

Data Analysis

ActivtyBase (Abase) version 5.4 from IDBS was used for the data processing and analysis. Individual Abase protocols and templates (HTS) were developed for each screening stage. All curve fitting was undertaken using XLFit version 4.2 from IDBS. A 4 Parameter Logistic dose response curve was utilised using XLFit 4.2 Model 205. Database querying and report creation was undertaken using SARgen version 5.4 from IDBS.
